# Thriving when living with chronic pain: A qualitative evidence synthesis of individuals' experiences

**DOI:** 10.1111/bjhp.70000

**Published:** 2025-06-20

**Authors:** Helena Widdrington, Charlotte Krahé, Katie Herron, Kimberley Smith, Mary Gemma Cherry

**Affiliations:** ^1^ Department of Primary Care and Mental Health University of Liverpool Liverpool UK; ^2^ School of Psychology Liverpool John Moores University Liverpool UK; ^3^ The Walton Centre NHS Foundation Trust Liverpool UK; ^4^ Department of Psychology University of Liverpool Liverpool UK

**Keywords:** acceptance, chronic pain, living well, PERMA, positive psychology, thriving

## Abstract

**Introduction:**

Research has begun to focus on positive adjustment to, and aspects of, living with chronic pain, which aligns with a positive psychology approach. This systematic review aimed to synthesize available qualitative data to understand the characteristics and approaches that enable people to thrive whilst living with chronic pain.

**Methods:**

Five electronic databases (MEDLINE, PsycINFO, CINAHL Plus, Scopus and ProQuest) were searched from their inception until January 2024 using a combination of terms for ‘chronic pain’, ‘thriving’ and ‘qualitative methods’. Inclusion criteria stipulated qualitative data pertaining to facilitators, barriers and inequalities in experiences of thriving in adults living with chronic pain (without a clear underlying cause). The methodological quality of studies was assessed using the Critical Appraisal Skills Programme Tool. Data were analysed using thematic synthesis, with GRADE‐CERQual used to determine confidence in the evidence.

**Results:**

In total, 4162 studies were screened; 17 were included in the qualitative synthesis. Thematic synthesis yielded four overarching themes: (i) attitudes towards pain and its impact on life; (ii) behavioural strategies and openness to trying new things; (iii) perceiving love, support and connection with others; and (iv) ascribing meaning to life alongside pain. GRADE‐CERQual indicated moderate confidence in findings.

**Discussion:**

Findings align with Seligman's five pillars of well‐being (PERMA) model and indicate clinical implications for supporting patients to thrive alongside pain.

## BACKGROUND

Chronic pain is defined by the International Association for the Study of Pain (IASP) for the International Classification of Diseases (ICD‐11) as any persistent bodily pain that has been present for 3 months or longer (International Association for the Study of Pain, 2020). Chronic pain is highly prevalent across the world, with data from 2016 suggesting that it is one of the leading causes of disease and disability globally (Vos et al., 2017). In the United Kingdom (UK), between one third to one half of the population is affected by chronic pain, with 12% of the population of England identifying chronic pain as high‐impact and disruptive to daily activities (Fayaz et al., 2016). The prevalence of chronic primary pain (i.e., pain that is not better accounted for by another diagnosis such as cancer) is estimated to be 1%–6% in England (National Institute for Health and Care Excellence [NICE], 2021a).

A deficit paradigm—that is, an approach that focuses on problems or negative outcomes—has predominated in the field of chronic pain research. Chronic pain has been associated with a range of negative outcomes including anxiety (Asmundson & Katz, 2009; Lerman et al., 2015), depression (Angst et al., 2020; Fishbain et al., 1997), sleep disturbances (Sun et al., 2021), decline in cognitive processes (Rouch et al., 2021), impairment of sexual function (Flegge et al., 2023), poorer quality of life (Hadi et al., 2019), pain‐related disability (Meulders, 2019) and increased suicide risk and suicidality (Petrosky et al., 2018; Tang & Crane, 2006). In addition, several meta‐analyses have examined psychological factors associated with, or predictive of, difficulty managing chronic pain (Georgopoulos et al., 2019; Martinez‐Calderon et al., 2019, 2020; Rogers & Farris, 2022; Zale et al., 2013). Findings indicated that higher pain hypersensitivity is predictive of reported pain‐related disability and negative affect (Georgopoulos et al., 2019), with the risk of onset and persistence of pain increased by psychological distress (i.e., depression and anxiety) and fear (Martinez‐Calderon et al., 2020). Whilst NICE (2021b) recommend cognitive behavioural therapy (CBT) as a non‐pharmacological approach to help people manage distress associated with/in the context of chronic pain, this approach is rooted in this deficit paradigm, with a focus on identifying and modifying cognitive and behavioural factors contributing to the development and maintenance of a presenting difficulty (Dobson & Dozois, 2021; Nezu et al., 2004). However, the efficacy of CBT for alleviating distress in chronic pain is low to moderate (de Williams et al., 2020). Incorporation of positive psychology approaches (in combination with current dominant deficit approaches such as CBT) has the potential to extend clinicians' capacity to provide support (Johnson & Wood, 2017; Wood & Tarrier, 2010) and help individuals live well with chronic pain.

Whilst comparatively less research has focused on positive adjustment to, and outcomes within chronic pain, this area is beginning to receive more attention. A recent meta‐analysis found that higher levels of positive affect are associated with decreased pain severity in individuals living with chronic primary pain (a small but significant effect size; Ong et al., 2020). A further review exploring psychological factors associated with the risk of onset and persistence of musculoskeletal pain found *‘protective’* or *‘positive’* associations that reduced these risks, such as self‐efficacy beliefs and positive beliefs regarding recovery (Martinez‐Calderon et al., 2020).

Exploring positive adjustment to living with chronic pain aligns well with a positive psychology approach (Seligman, 1992). Pathology‐led psychology has been argued to neglect viewing humans as proactive and self‐determined beings; a positive psychology approach instead focuses on recognizing personal strengths, connecting with others and developing gratitude and awareness despite difficulties (Seligman & Csikszentmihalyi, 2000). Whilst the term *‘positive psychology’* serves as an umbrella term that encompasses several key concepts (Schrank et al., 2014), Seligman (1992) identified five pillars of well‐being integral to a positive psychology approach, which he organized into the mnemonic ‘PERMA’: positive emotion, engagement (i.e., in activities that pull on personal strengths); relationships (i.e., positive, respectful and understanding relationships); meaning (i.e., identifying positive meaning in everyday life); and accomplishment (i.e., achievements, big or small, that help us feel progress; Seligman, 2011). A positive psychology approach focuses on these five elements, drawing on personal strengths and resources in the prevention and/or treatment of difficulties, such as living with chronic pain, to help individuals, families and communities to *‘thrive’* despite difficulties (Park et al., 2016).

**FIGURE 1:**
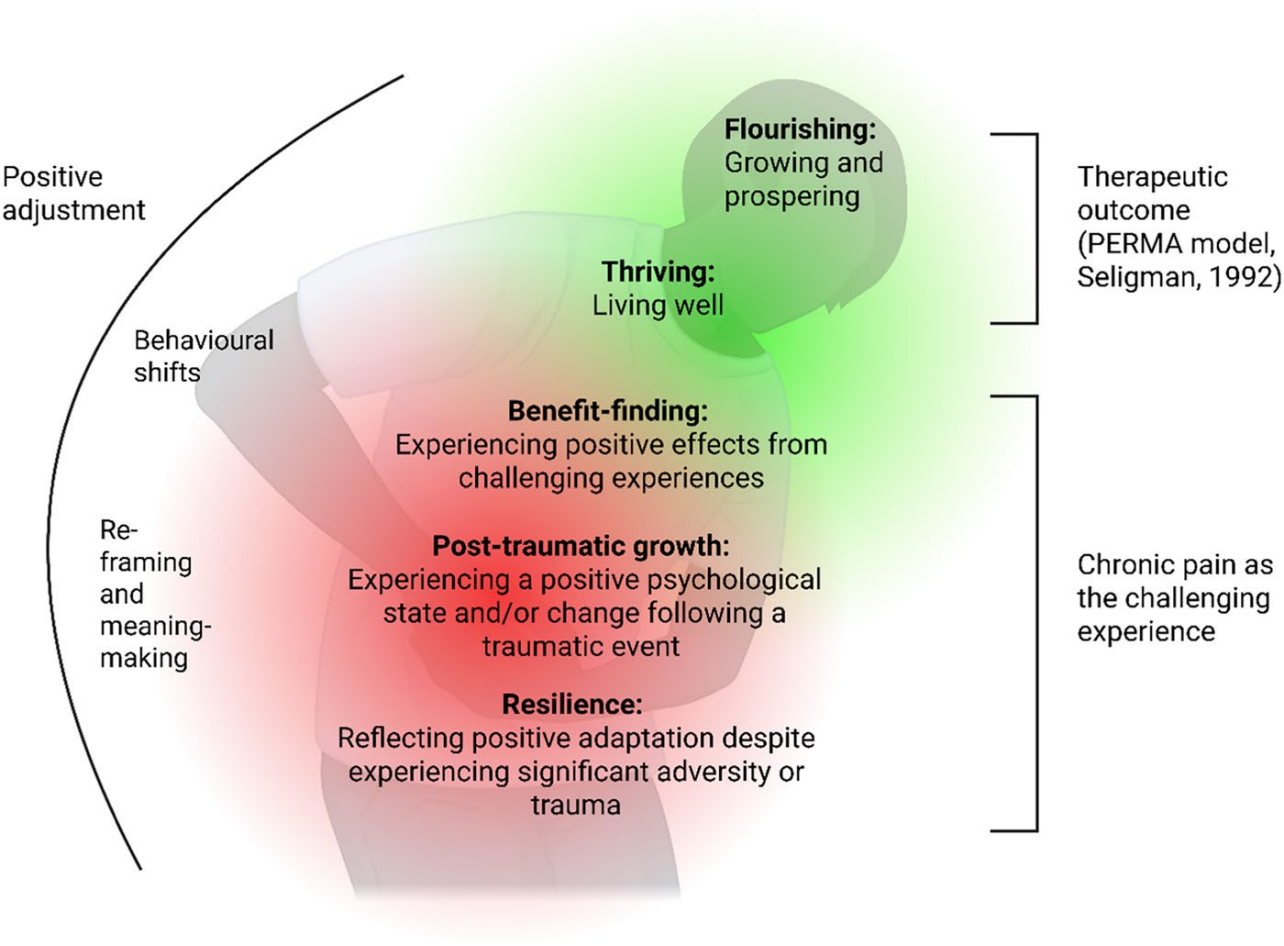
Conceptual map of positive adjustment to chronic pain, drawing on definitions of flourishing (VanderWeele et al., 2019), thriving (Brown et al., 2017) benefit‐finding (Cassidy et al., 2014; Helgeson et al., 2006), post‐traumatic growth (Tedeschi et al., 2018) and resilience (Seligman, 1992). No hierarchy between concepts is intended. Created in https://BioRender.com/dsr824w.

To design effective and acceptable interventions rooted in a positive psychology paradigm, it is important to identify and understand what helps or supports individuals to thrive. In positive psychology, thriving is seen as a therapeutic outcome, closely linked to the concept of ‘flourishing’. In this review, we consider thriving as maintaining positive well‐being in the context of the difficulties posed by living with chronic pain—where pain is the primary concern. In capturing living well in the context of adversity, thriving also overlaps with concepts such as resilience, benefit‐finding and post‐traumatic growth. To conceptualize the links between the described concepts, Figure 1 groups the personal characteristics and approaches that the literature suggests support thriving and flourishing as a therapeutic outcome. Resilience, post‐traumatic growth and benefit‐finding are considered here in relation to chronic pain as the challenge or adverse or traumatic event, and include both cognitive processes, such as reframing, and behavioural shifts that lead to growth. Thriving and flourishing are therapeutic outcomes in the PERMA model, indicating subjective well‐being and are not necessarily tied to having experienced adversity (Brown et al., 2017), though we here conceptualize them as reflecting positive adjustment to, and living well with, chronic pain. Thriving and flourishing encompass growth processes and are conceptually linked to benefit‐finding, post‐traumatic growth and resilience, for example through improving functioning; flourishing is more commonly relating to psychosocial well‐being whilst thriving encompasses both psychosocial and physical states (Brown et al., 2017). Thus, we adopt the term ‘thriving’ throughout the review to represent these interlinked—though not identical—concepts and to allow us to capture primary literature on factors enabling positive adjustment to living with chronic pain.

Quantitative studies indicate that people living with chronic pain can and do thrive (e.g., Parsons et al., 2022; Sirois & Hirsch, 2013) and that thriving is linked to more positive pain‐related outcomes (e.g., intensity and disability; Kinderdietz, 2012; Palit et al., 2020), reduced distress (Currier et al., 2009; Min et al., 2014; Sirois & Hirsch, 2013), greater positive affect (Kinderdietz, 2012) and enhanced well‐being (e.g., greater appreciation for life and better relationships; Abraído‐Lanza et al., 1998; Dirik & Karanci, 2008; Purc‐Stephenson, 2014). Moreover, some quantitative research has examined how ‘resilient’ individuals living with chronic pain differ from those deemed by the researchers to be ‘non‐resilient’ in their coping style, pain attitudes and beliefs, and positive and negative social responses to pain (Karoly & Ruehlman, 2006).

Qualitative research provides rich, detailed descriptions of complex phenomena, considering factors such as personal meaning and social context. For example, in relation to chronic pain, Lennox Thompson et al. (2020) found that thriving involves resolving a disruption to one's self‐coherence by making sense of pain, deciding to see oneself as a person, not a patient and engaging with life flexibly. Richardson et al. (2014) similarly identified flexibility, understanding of pain, and motivation to keep going as key factors shaping resilience whilst living with chronic pain. Understanding people's lived experience is key to designing and implementing effective and acceptable interventions, and qualitative insights into what helps people to thrive whilst living with chronic pain can give a rich starting point for therapeutic intervention. However, qualitative evidence on thriving in chronic pain has not yet been systematically synthesized. Using our conceptualization of thriving and including associated concepts (see Figure 1), we thus aimed to conduct a broad and inclusive qualitative evidence synthesis to explore which personal characteristics and approaches enable people to thrive whilst living with chronic pain.

## METHOD

The conduct and reporting of the review are in accordance with Preferred Reporting Items for Systematic Reviews and Meta‐Analyses (PRISMA) guidance. The review protocol can be accessed via PROSPERO database (CRD42024501605) where it was registered prior to completing any searches.

### Search strategy

Following initial scoping searches, four electronic databases (MEDLINE, PsycINFO, CINAHL Plus and Scopus) were searched for relevant literature from their inception until January 2024. To ensure a comprehensive search of grey literature, ProQuest was also searched for theses/dissertations from inception to January 2024. Hand searching was also conducted via Google Scholar and examination of the reference lists of included studies and relevant systematic reviews. Search syntax was finalized in PsycINFO (Table 1) and adapted for each electronic database.

**TABLE 1:** Search terms used for PsycINFO.

Domain		Criteria	Search term
P	Population	Adults living with chronic pain	“Chronic pain” OR “persistent pain” OR “long‐term pain” OR “recurrent pain” OR “Chronic primary pain”
			AND
I	Interest	Any experiences relating to thriving	Flourish* OR “Positive Change” OR “benefit find*” OR “benefit‐find*” OR “Post Traumatic Growth” OR “Post‐Traumatic Growth” OR “Adversarial Growth” OR “posttraumatic growth” OR PTG or “vicarious PTG” OR “Silver Lining” OR “Silver‐Lining” OR resilien* OR thriv* OR grow OR “positive adjustment” OR “make meaning” or “find* meaning”
			AND
Co	Context	Any study that has qualitative data	qualitative* OR “qualitative research” OR “focus group*” OR “interview*” OR “grounded theory” OR “IPA” OR “thematic analysis” OR Phemenolog* OR “mixed method*” OR ethnographic OR epistemology OR “semi‐structured” OR semistructured OR unstructured OR experience OR view OR perspective

### Study screening and selection

All identified citations were exported to EndNote X9 (2018); following removal of duplicates, titles and abstracts of the remaining studies were imported to Rayyan (an online review software platform; Ouzzani et al., 2016) and screened against the eligibility criteria. Studies that did not meet these criteria were excluded. The full text of potentially relevant papers was then screened to determine eligibility. Screening was completed independently by the primary researcher (HW) and a second reviewer (KS), who screened approximately 37% of titles/abstracts and titles and 20% of full‐text papers. Disagreements were discussed until consensus was reached, with the wider team consulted if required.

Studies were included if they were empirical, peer‐reviewed research or grey literature, were written in English and reported qualitative data in adult (aged 18+ years) participants. As our focus was on pain as the overarching difficulty, we included only studies focusing on people living with chronic primary pain or arthritic pain. Chronic primary pain describes pain lasting longer than 3 months with no clear underlying secondary cause (e.g., health conditions such as cancer). Examples include fibromyalgia, complex regional pain syndrome, chronic migraine, irritable bowel syndrome and chronic low back pain (Nicholas et al., 2019). We included arthritic pain because it may persist after inflammation is controlled, indicating pain mechanisms are driven by changes in the central nervous system leading to neuroplastic changes (Bushnell et al., 2013) and central sensitization (Woolf, 2011), similar to chronic primary pain. Other secondary causes were excluded, as we reasoned that thriving might look different when living with a life‐limiting condition such as cancer. Lastly, studies were included only if they made specific reference to facilitators, barriers and inequalities in thriving in chronic pain.

Studies were excluded if they reported: (i) quantitative data only; (ii) mixed‐methods data from which qualitative data could not be extracted; (iii) data from adults living with chronic pain that could not be separated from adults with broader health conditions, or those with secondary chronic pain; (iv) general experiences of individuals living with chronic pain rather than in regard to thriving; (v) data from participants under the age of 18, and/or individuals without chronic pain diagnoses; and/or (vi) were not accessible or written in English.

### Assessment of risk of bias

Included papers were assessed independently by two reviewers (HW and KS) for risk of bias using the Critical Appraisal Skills Programme (CASP, 2018) checklist for qualitative studies. Disagreement was resolved through consensus or consultation with the wider research team (MGC, CK, KH). This tool asks reviewers to select either a response of ‘Yes’, ‘No’ or ‘Can't Tell’ to 10 questions exploring the validity of results, appropriateness, scientific rigour and usefulness of the studies. In line with the Centre for Reviews and Dissemination [CRD] (2009), studies were not excluded where risk of bias was indicated; however, risk of bias was considered when interpreting results. The identified reviewers were not blinded to the authors, institutions, or journals of included studies.

### Data extraction

The primary researcher (HW) developed a data extraction form informed by guidance from Noyes et al. (2020) and the principles of thematic synthesis (Thomas & Harden, 2008). Participant and study characteristics, analysis type, themes, quotations and authors' interpretations were extracted by HW and cross‐checked by an independent reviewer (KS). The following data were extracted: author, discipline of the author, year of publication, type of research (grey literature/peer reviewed), study design and aims, demographic characteristics of participants (age, gender, ethnicity, pain type, duration of chronic pain), qualitative analysis method/approach, main findings, themes, quotations and interpretations of results.

### Data synthesis

Data were synthesized using thematic synthesis (Thomas & Harden, 2008). This was conducted by the primary researcher (HW) and reviewed by the wider research team. During the first stage, data familiarization, the primary researcher familiarized themselves with individual studies and the extracted data from each study; these were referred to throughout the synthesis, whilst also capturing the context of the study in which the data were generated. Considering the wider context of studies helped to reduce the risk of placing preconceived ideas on expected findings (Thomas & Harden, 2008). The studies were then imported to NVivo, whereby the primary researcher (HW) coded data line‐by‐line to identify patterns. Codes were generated that explained large parts/patterns of the data, following which they were reviewed, considering meaning and contextual factors across the different studies and aiming towards the generation of ‘data‐driven’ descriptive themes (Thomas & Harden, 2008). For the final stage of synthesis, ‘going beyond’ the aggregated findings from the primary studies (Thomas & Harden, 2008), the primary researcher inductively developed analytical themes. The synthesis process and final themes were reviewed by the wider research team and refined accordingly. Throughout the process of refining the analysis, the lead researcher regularly circled back to the original studies to ensure themes were grounded in the context of each study. Finally, to explore coherence and to assess how much confidence to place in the qualitative synthesis, the GRADE‐CERQual approach (Lewin et al., 2018) was used. This approach is designed to improve the quality and trustworthiness of qualitative research findings by systematically evaluating confidence in them. Each finding was assessed using the stipulated four criteria (i.e., methodological limitations, coherence, adequacy and relevance) to determine the strength of the qualitative evidence.

Narrative summaries of each theme and a thematic diagram highlighting key codes are presented in the Results section to provide transparency of the synthesis completed. A sample of original quotations from studies for each of the themes are interwoven within the results to demonstrate participants' voices and transparency of data. GRADE‐CERQual findings are presented for each theme.

## RESULTS

**FIGURE 2:**
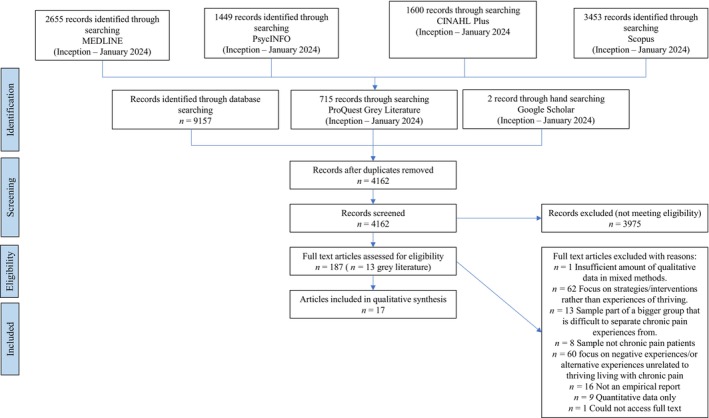
PRISMA diagram illustrating the screening process.

Figure 2 shows the flow of studies through the review. In total, 9872 studies were identified through database searching, resulting in 4162 unique citations to be screened. The full text of 187 were subsequently screened, resulting in the inclusion of 17 studies.

### Characteristics of included studies

Table 2 shows the characteristics of the included studies. All were published between 1999 and 2023 and conducted in the Unites States of America (*n =* 8), United Kingdom (*n =* 3), Australia (*n =* 2), India (*n =* 1), Sweden (*n =* 2) and New Zealand (*n =* 1). Study designs included mixed methods (*n* = 2), sequential mixed methods (*n* = 1), longitudinal mixed methods (*n* = 1), autoethnographic (*n* = 1), grounded theory (*n* = 2), qualitative (*n* = 8) and longitudinal qualitative (*n* = 2). Qualitative data were gathered through methods including semi‐structured interviews and in‐depth interviews. Data were analysed using a variety of methods/approaches, including thematic analysis, narrative analysis, interpretive phenomenological analysis (IPA), grounded theory, Qualitative Analysis Guide of Leuven (QUALOG), heuristic inquiry, insider research and autoethnography.

**TABLE 2:** Study and participant characteristics.

Author and year	Country	Study type and design	Recruitment method	Participant characteristics				Pain‐related characteristics		Analysis
				*N*	Age (years), mean (*SD*)	Male, *n* (%)	Ethnicity	Type, *n* (%)	Mean duration, years (range)	Method of analysis
Bergsten et al. (2011)	Sweden	Qualitative; peer‐reviewed	Nurse involved in care	16	Women: 62 (range: 28–82) Men: 61 (range: 42–70)	6 (37.5)	Swedish	RA	14 (2–42)	Grounded Theory
Chavare and Natu (2020)	India	Qualitative; peer‐reviewed	Private clinical and personal contacts	8	NR (Range: 30–75)	0	Indian	RA	NR (3–55)	Thematic Analysis
Danoff‐Burg and Revenson (2005)	USA	Longitudinal mixed methods; peer‐reviewed	Patient registry at hospital	136	58 (14.05)	25 (18.4)	NR	RA	16	Thematic Analysis
Hernandez (2022)	USA	Qualitative; not peer‐reviewed	Purposive and judgemental	8	NR (Range: 34–62	0	NR	Chronic Pain	NR	Heuristic Inquiry
Landgren et al. (2020)	Sweden	Qualitative longitudinal; peer‐reviewed	Purposive sampling	T1: 31 T2:22	T1: NR (Range: 38–80) T2: NR (Range: 42–81)	T1: 9 (29) T2: 7 (31.8)	NR	RA	T1: (.3–.9) T2:(1–1.9)	T1 and T2 compared through Qualitative Analysis Guide of Leuven (QUALOG)
Lennox Thompson et al. (2020)	New Zealand	Grounded theory; peer‐reviewed	Purposive via social media	17	44.53 (13.56)	8 (47)	NR	RA: 3 (17) Psoriatic Arthritis: 2 (11.8) Osteoarthritis: 5 (29.4) Fibromyalgia: 3 (17.7) Other: 9 (52.9)	13.11	Grounded Theory
Loffer (1999)	USA	Qualitative; not peer‐reviewed	NR	9	NR (Range: 30s‐40s)	0	NR	RA	(9–22)	Insider Research
Owens et al. (2016)	USA	Mixed methods; peer‐reviewed	Convenience sampling via newspaper advertisement	80	53.6 (12.5)	20 (25)	White 74 (92.5%) Black 4 (5%) Hispanic 2 (2.5%)	Musculoskeletal: 43 (53.8); Neuropathic: 17 (21.3); Musculoskeletal and Neuropathic: 10 (25)	17.7	Informed by Calhoun and Tedeschi's Posttraumatic Growth Model and Ardelt's 3 D‐WM
Richards (2023)	UK	Autoethnography; peer‐ reviewed	NA	1	NR	0	NR	Chronic Pain	NR	Autoethnography
Richardson et al. (2014)	UK	Qualitative longitudinal; peer‐reviewed	Ongoing cohort study (NorStOP study)	27	NR (Range: 55–90)	NR	NR	Osteoarthritis/Chronic Joint Pain:	NR	Thematic Analysis
Rolbiecki et al. (2017)	USA	Qualitative; peer‐reviewed	Outpatient clinic, social media, personal contacts	12	NR (Range: early 20s‐early 80s	3 (25)	Caucasian	Chronic Pain	NR (.6–.8 to 1)	Thematic Analysis
Saul (2015)	USA	Mixed methods; not peer‐reviewed	Invitation following quantitative study	10	NR (Range: 65–75)	NR	NR	Chronic Back Injury: 5 (50)	NR	Thematic Analysis
Shaw et al. (2020)	USA	Qualitative; peer‐reviewed	FORWARD (The National Databank for Rheumatic Diseases, a US‐based registry of patients with rheumatic diseases) enrolees invited	18	63.6 (Range: 27.4–80.3)	5 (28)	White 16 (89%) African American 1 (5%) Hispanic 1 (5%)	RA	21.1 (5.3–41)	Narrative
Sheedy et al. (2017)	Australia	Sequential mixed methods; peer‐reviewed	General practice	6	47 (Range: 33–65	1 (17)	NR	Fibromyalgia: 3 (50) Low Back Pain and Fibromyalgia: 2 (33) Osteoarthritis and Fibromyalgia: 1 (17)	5.58 (1.5–10)	Thematic Analysis
Swift et al. (2002)	UK	Qualitative; peer‐reviewed	Convenience sample	5	NR (Range: 63–89)	0	NR	Osteoarthritis	NR (2–16)	IPA
Taylor (2021)	USA	Qualitative; not peer‐reviewed	Email and social media	6	47.5 (Range: 31–73	1 (16.7)	NR	Fibromyalgia: 1 (17) RA: 1 (17) Pain in more than one part of body: 4 (66)	14.3 (3–30)	IPA
West et al. (2012)	Australia	Qualitative; peer‐reviewed	Part of larger study	10	NR (Range: 26–54)	4 (40)	NR	Chronic Pain	NR (4–30)	Thematic Analysis

Abbreviations: IPA, interpretative phenomenological analysis; NR, not reported; RA, rheumatoid arthritis; T1, timepoint 1; T2, timepoint 2.

The total number of participants ranged from 1 to 136, with most identifying as female. Ages ranged from 20 to 90 years old, as presented in Table 2. Only five studies reported ethnicity data, of which 84.91% of the participant were White. Approximately 60.44% of the sample lived with Rheumatoid Arthritis, with approximately 10% experiencing osteoarthritis/chronic joint pain and 5% experiencing chronic pain. The average duration of chronic pain ranged from 6 months to 55 years (average = 12.91 years, standard deviation [*SD*] = 6.34 years).

### Assessment of risk of bias

The results of the risk of bias assessment are depicted in Table 3. Overall, results indicated that studies presented with some risk of bias, with few studies meeting all the criteria as stipulated in the tool (CASP, 2018). All studies used appropriate qualitative methodology and all except Loffer (1999) clearly outlined their aims. The criteria for the appropriateness of recruitment strategy, data being collected in a way to address the research question, and having a clear statement of findings was met by all studies except Richards (2023).

**TABLE 3:** Risk of bias assessment scores.

Author(s) and publication year	Was there a clear statement of the aims of the research?	Is a qualitative methodology appropriate?	Was the research design appropriate to address the aims of the research?	Was the recruitment strategy appropriate to the aims of the research?	Was the data collected in a way that addressed the research issue?	Has the relationship between researcher and participants been adequately considered?	Have ethical issues been taken into consideration?	Was the data analysis sufficiently rigorous?	Is there a clear statement of findings?
Bergsten et al. (2011)	Y	Y	Y	Y	Y	N	Y	N	Y
Chavare and Natu (2020)	Y	Y	N	Y	Y	N	Y	Y	Y
Danoff‐Burg and Revenson (2005)	Y	Y	Y	Y	Y	Y	N	Y	Y
Hernandez (2022)	Y	Y	Y	Y	Y	Y	Y	Y	Y
Landgren et al. (2020)	Y	Y	Y	Y	Y	Y	Y	Y	Y
Lennox Thompson et al. (2020)	Y	Y	Y	Y	Y	CT	Y	Y	Y
Loffer (1999)	CT	Y	Y	Y	Y	Y	CT	CT	Y
Owens et al. (2016)	Y	Y	Y	Y	Y	Y	Y	Y	Y
Richards (2023)	Y	Y	N	N	N	N	N	N	N
Richardson et al. (2014)	Y	Y	Y	Y	Y	Y	Y	Y	Y
Rolbiecki et al. (2017)	Y	Y	N	Y	Y	Y	Y	N	Y
Saul (2015)	Y	Y	Y	Y	Y	Y	Y	Y	Y
Shaw et al. (2020)	Y	Y	Y	Y	Y	Y	Y	Y	Y
Sheedy et al. (2017)	Y	Y	Y	Y	Y	N	Y	Y	Y
Swift et al. (2002)	Y	Y	N	Y	Y	Y	Y	Y	Y
Taylor (2021)	Y	Y	Y	Y	Y	Y	N	Y	Y
West et al. (2012)	Y	Y	Y	Y	Y	Y	Y	Y	Y

Abbreviations: CT, cannot tell; N, no; Y, yes.

Across the studies, limitations included insufficient documentation on the relationship between researcher and participants. Specifically, four studies neglected to critically reflect and examine the authors' roles, and the potential bias introduced by this aspect, or did so insufficiently (Bergsten et al., 2011; Chavare & Natu, 2020; Richards, 2023; Sheedy et al., 2017). Four studies did not sufficiently justify their choice of method (Chavare & Natu, 2020; Richards, 2023; Rolbiecki et al., 2017; Swift et al., 2002). Richards (2023) only met two out of the nine criteria. This was due to this study employing autoethnographic methodology (i.e., reflecting on the author's personal experiences). Four of the studies were not peer‐reviewed (Hernandez, 2022; Loffer, 1999; Saul, 2015; Taylor, 2021), but despite this, performed relatively well on the CASP assessment. Loffer (1999) did not report whether ethical issues had been considered, if data were scientifically rigorous, and did not report the aims of the study.

### Qualitative synthesis

**FIGURE 3:**
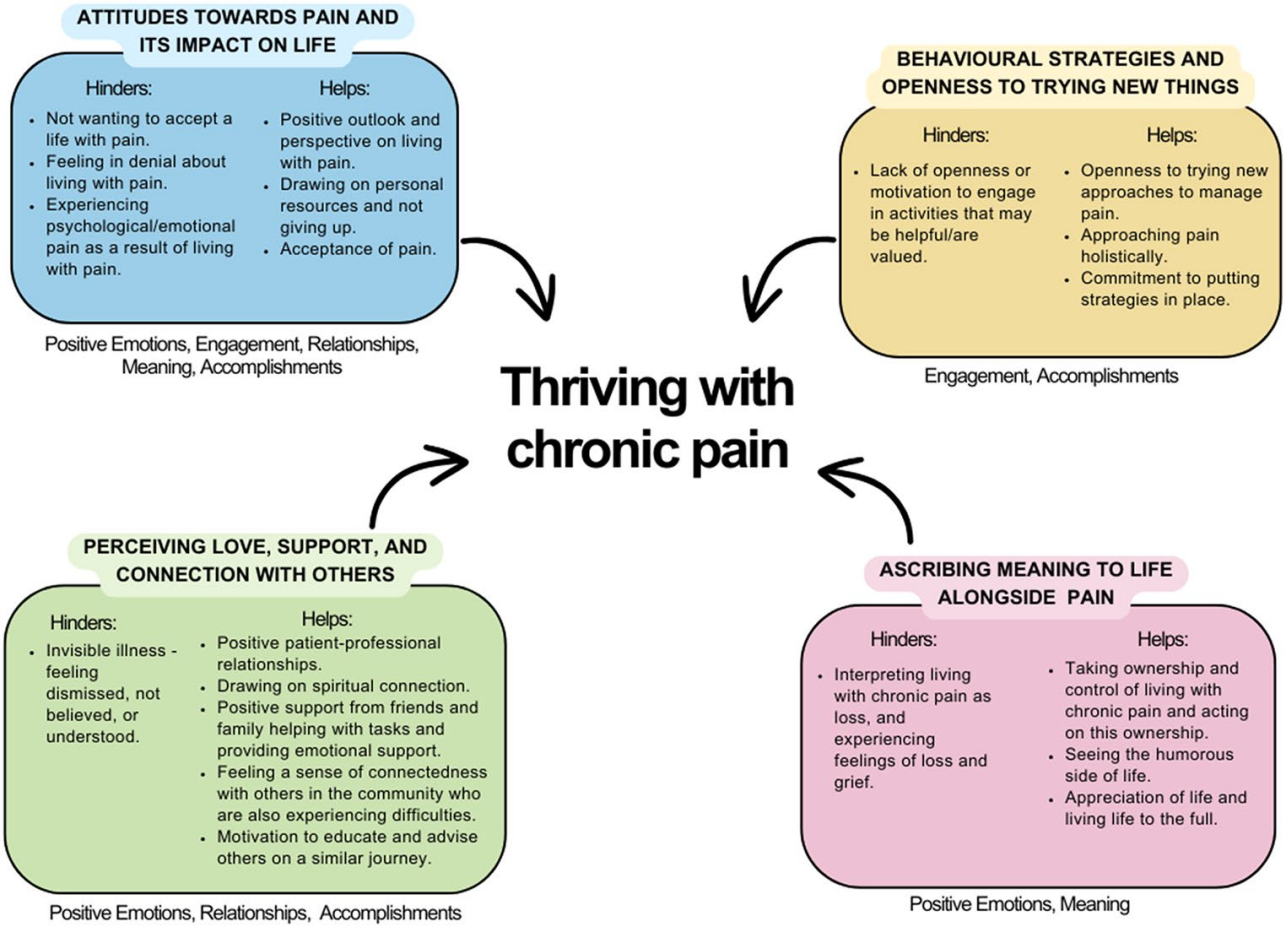
Visual summary of the findings. Notes under each theme indicate the PERMA pillars with which each theme best aligns.

Four overarching themes were identified, as shown in Figure 3, which were each deemed to be important in helping an individual to thrive with chronic pain: (i) attitudes towards pain and its impact on life; (ii) behavioural strategies and openness to trying new things; (iii) perceiving love, support and connection with others; and (iv) ascribing meaning to life alongside pain. The GRADE‐CERQual results (see Table 4) indicated moderate confidence that all findings are a reasonable representation of what the review set out to explore (i.e., thriving in individuals living with chronic pain).

**TABLE 4:** GRADE‐CERQual findings.

#	Summarized review finding	GRADE‐CERQual assessment of confidence	Explanation of GRADE‐CERQual assessment	References
**Theme 1: Attitudes towards pain and its impact on life**				
1	In 17 out of 17 studies, participants discussed how their attitudes towards pain (i.e., acceptance, outlook, drawing on personal resources) and its impact on life either helped or hindered them to thrive	Moderate confidence	Moderate concerns regarding methodological limitations, Minor concerns regarding coherence, No/Very minor concerns regarding adequacy, and No/Very minor concerns regarding relevance	Saul (2015), Loffer (1999), Taylor (2021), Hernandez (2022), Chavare and Natu (2020), Sheedy et al. (2017), Richardson et al. (2014), Danoff‐Burg and Revenson (2005), Bergsten et al. (2011), Richards (2023), Lennox Thompson et al. (2020), West et al. (2012), Swift et al. (2002), Shaw et al. (2020), Rolbiecki et al. (2017), Owens et al. (2016), Landgren et al. (2020)
**Theme 2: Behavioural strategies and openness to trying new things**				
2	In 16 out of 17 papers, participants discussed how being open to trying new approaches to manage pain and being committed to put these in place, can help them thrive	Moderate confidence	Moderate concerns regarding methodological limitations, Minor concerns regarding coherence, No/Very minor concerns regarding adequacy, and Minor concerns regarding relevance	Saul (2015), Loffer (1999), Taylor (2021), Hernandez (2022), Chavare and Natu (2020), Sheedy et al. (2017), Richardson et al. (2014), Bergsten et al. (2011), Richards (2023), Lennox Thompson et al. (2020), West et al. (2012), Swift et al. (2002), Shaw et al. (2020), Rolbiecki et al. (2017), Owens et al. (2016), Landgren et al. (2020)
**Theme 3: Perceiving love, support and connection with others**				
3	In 17 out of 17 papers, participants discussed how perceiving help, love and support from others (professionals and non‐professionals), and connection with others (helping others helps them), can enable them to thrive when living with chronic pain	Moderate confidence	Moderate concerns regarding methodological limitations, minor concerns regarding coherence, no/very minor concerns regarding adequacy and minor concerns regarding relevance	Saul (2015), Loffer (1999), Taylor (2021), Hernandez (2022), Chavare and Natu (2020), Sheedy et al. (2017), Richardson et al. (2014), Danoff‐Burg and Revenson (2005), Bergsten et al. (2011), Richards (2023), Lennox Thompson et al. (2020), West et al. (2012), Swift et al. (2002), Shaw et al. (2020), Rolbiecki et al. (2017), Owens et al. (2016), Landgren et al. (2020)
**Theme 4: Ascribing meaning to life alongside pain**				
4	In 15 out of 17 papers, participants discussed taking ownership of living with chronic pain and acting on this as moving towards thriving	Moderate confidence	Moderate concerns regarding methodological limitations, minor concerns regarding coherence, no/very minor concerns regarding adequacy and minor concerns regarding relevance	Saul (2015), Loffer (1999), Taylor (2021), Hernandez (2022), Chavare and Natu (2020), Sheedy et al. (2017), Richardson et al. (2014), Danoff‐Burg and Revenson (2005), Bergsten et al. (2011), Richards (2023), Lennox Thompson et al. (2020), West et al. (2012), Swift et al. (2002), Shaw et al. (2020), Rolbiecki et al. (2017), Owens et al. (2016), Landgren et al. (2020)

### Main themes

#### Attitudes towards pain and its impact on life

The attitudes held by individuals towards their pain appeared to be an important factor in determining whether or not they thrived. Factors that helped an individual to thrive included having a positive outlook and perspective on living with pain, drawing on personal resources, not ‘giving up’ when faced with pain and accepting pain as present in their lives, as shown in Figure 3. By contrast, factors that appeared to hinder thriving included an unwillingness to accept a life with pain, denial of the existence of pain and experiencing psychological or emotional pain (i.e., anxiety and/or depression).

Participants within the included studies discussed having a positive outlook and perspective regarding pain, seeing this as something that they had a choice over. They reported they felt able to choose to perceive their life with pain as positive, which helped them to live well with the condition. For example, a participant in West et al. (2012) reported,

I have a positive attitude… I'm trying to get a life and live a life, not live an existence and that's why I keep going… (West et al., 2012)

Participants from the study conducted in India indicated that they chose to have a sense of optimism regarding living with chronic pain, which included positively rephrasing negative thoughts, holding expectations that outcomes in future will be positive and having confidence in their capacity to fulfil their responsibilities and look at the positive aspects of their life: “I feel blessed that I don't have any kind of deformities. I do not see any limitations to lead normal life.” (Chavare & Natu, 2020). Participants discussed how having a positive attitude kept them going, looking forward, and living the life that they wanted. A participant in Sheedy et al. (2017) stated: “I'll try and accept that I've got this banner now and I sit under it but I can … move beyond it… I'm just going to have to make positive, informed choices about how I will treat the condition and in so doing be able to take on more normal things again.” (Sheedy et al., 2017). This quote highlights the role of acceptance of pain as being key to navigating living with the condition and thriving. Acceptance enabled participants to shift their attitude from fighting and resistance, thus giving them more energy to live the life that they wanted, instead of focusing on pushing the pain away:

You know what's funny about the pain—it seems as though fighting the pain was such a big deal before. When I finally just accepted that I do have some pain, and I quit fighting it and I quit resisting it so much—then it wasn't as big an issue in my life. (Loffer, 1999)

By contrast, being ‘in denial’ and resisting pain took up valuable energy, space and time, thus hindering thriving. For example, participants from Taylor (2021) reported:

I think the denial would slip in more often when I had periods of time when I felt better: when I could leap out of bed and just go and go and go and not feel physically exhausted by it. Until the next time when, of course, I would have a flare, and then it was, Uh, oh, it's back.

I resisted life and that made my pain worse because I refused to accept it (Owens et al., 2016)

The act of trying to resist, push the pain away and struggling to accept the pain made the experience of pain worse for participants, and in turn hindered them from living well with the condition. Experiencing or focusing on the psychological or emotional pain that results from living with the condition also appeared to limit thriving. In one study, a participant reported that even being asked questions about the emotional difficulties related to pain triggered difficult feelings for them, indicating the emotional toll that living with pain can have:

I just don't like to think about it [how I'm doing]. If I wrote it down somewhere, and then had a really bad day, it would make me upset. (Richardson et al., 2014)

#### Behavioural strategies and openness to trying new things

Participants within the included studies were largely open to trying different strategies to identify those that worked for them. Being open, curious to explore and learn different approaches to manage pain helped participants to live well with the condition. Participants in the study conducted in India sought information about their pain on the internet or read books to gain scientific understanding; by contrast, participants within the remaining studies engaged in activities such as Yoga, Pilates and diet changes:

At age 25 I had arthritis that I'm very much linking to a lot of pain that I was going through in my marriage. I went to a naturopath who taught me how to do self‐massage, how to change my diet. I had a lot of success with reducing the pain through the natural practices. I was also happy I made the association between my emotions and pain. (Owens et al., 2016)

As part of being open to trying new strategies, participants discussed looking at their pain holistically, seeing it as a mind and body experience, which helped them to thrive with pain. Holistic patient care involves attending to patients' physical, psychological, spiritual and social needs. One participant suggested delivering more holistic patient care could help individuals to thrive:

One thing I wish would happen. Physicians deal with the medical part, and also the person. Yes, but there needs to be something additional. Someone. I don't know if a therapist should come in if a person wants, but maybe somebody that helps them understand what's going on better at first. (Hernandez, 2022)

This quote indicates that participants are seeking not just physical support for their pain, but care that helps them to understand what is happening in their mind as well as their body, with this in turn helping individuals to live well.

Being committed to use strategies, as appropriate, was also key in moving participants towards thriving with chronic pain. One participant discussed how this can mean not surrendering to the pain they experience, knowing that they will feel better if they continue moving despite being in pain:

I can't allow the illness to take me: coping, coping is what I do. Coping with the terrible feelings of what I will do even if I completely down because of the feeling on my body. Coping with moving on. I can't allow the illness to take over my body. I have to be stronger than the illness. I can't allow the illness to take me. (Saul, 2015)

Across all cultures, participants discussed a perseverance to engage in activities of personal value whilst living with the condition. They acknowledged that they are living with pain, and it might be there all the time, but they did not want that to come in the way of completing activities they valued. Participants who were able to move towards their values appeared to demonstrate flexibility in that they could adapt and find new ways to engage with their values despite being in pain. In the study conducted in India, participants discussed making adaptations to enable them to persevere with daily activities relating to them fulfilling a role (i.e., mother, daughter), as the following two quotes from Chavare and Natu (2020) illustrate:

In kitchen, I could not lift heavy utensils. Therefore, I started using light weighted utensils

Due to RA [Rheumatoid Arthritis], sometimes joints pain like anything. But at that time, it is like if I have decided to work then I have to do it. It is fine for me even though I get pain next day.

Participants in the remaining studies discussed continuing to do activities of value to them, including hobbies:

It was like I had to get back to my sport, or what's the point? Nothing was going to stop me once I started, it's what I love. (Lennox Thompson et al., 2020)

I was a swimmer before and still call myself a swimmer, although I'm not as crazy as I used to be. But that is still a part of who I am. (Loffer, 1999)

Shutting off from trying new activities or approaches, or lacking motivation to engage in potentially helpful approaches, appeared to hinder thriving. For instance, a participant from Taylor (2021) reported that they were unable to engage with recommended pain management activities due to anxiety: “She has recommended that I do yoga, physiotherapy, occupational therapy, and other things, but I stay at home because of my anxiety…” (Taylor, 2021). Some people reported difficulty in putting recommendations from professionals into place without the right support.

Participants also felt caught between wishing to do valued activities and experiencing pain interference; for example, one participant in Sheedy et al. (2017) reported: “It's not being able to … do the physical things the way that you have done them in the past, you try to do something, and you just get stopped by a wall of pain. So you don't do it, and then you don't do it again.” (Sheedy et al., 2017). They shared how they could get stuck in a cycle whereby disengaging from an activity made it more difficult to get back to doing that activity again. They reported being stopped by a “wall of pain”, which was a barrier to living well.

#### Perceiving love, support and connection with others

Perceiving practical and emotional support from others, as well as feeling a sense of connection to others going through similar difficulties, aided individuals to thrive when living with chronic pain. This took several forms, including positive patient–professional relationships, spiritual connection, positive support from friends and family and connection with others in the community also experiencing difficulties, as presented in Figure 3.

Participants reported they had confidence in their doctor and felt reassured by the knowledge and care that they deliver. For example, in the one study conducted in India, participants particularly stressed the importance of the patient–professional relationship in the initial stages of living with chronic pain (Chavare & Natu, 2020). Reliance on doctors' advice on aspects of care such as medication dosage was common, with participants relying less on doctors over time as they began to learn from their own experience. For example, a participant reported:

My rheumatologist said positive things that you have to take medicines regularly then you will be fine. My doctor guided me a lot that he said just keep on doing your daily chores. If doctor says positive things your optimism increases. (Chavare & Natu, 2020)

By contrast, the remaining studies found that, despite patient–professional relationships being important in helping individuals thrive when living with pain, positive experiences were not ubiquitous. For example, one participant in Hernandez (2022) discussed changing doctors to see a professional who took a more holistic approach: “I think is a good thing to experiment if you have the right support, I see a holistic doctor here as well and he's really good.” (Hernandez, 2022).

Participants also discussed gaining strength from spiritual connections. In the study conducted in India (Chavare & Natu, 2020), participants discussed finding solace in praying and reading philosophical teachings of saints. Engaging in these activities helped participants to gain positive energy, strength and support. Studies conducted in Western countries made little reference to reading philosophical teachings; rather, participants reported feeling a sense of calmness and connection through engaging in spiritual connection and praying. Quotations such as this one from Rolbiecki et al. (2017) were common throughout the data: “My faith helps me make it through.” (Rolbiecki et al., 2017). Similarly, a participant in Owens et al. (2016) stated:

One thing that helps me with my physical pain is to start the day with the, the love of the Lord. There's actually a physical response. Stress causes inflammation, more pain, it's a nasty cycle. If I stay in a more relaxed mindset. I've got calmer hormones, positive endorphins pumping through my body. That helps my physical pain, less inflammation, less pain, less grumpy…if we stay positive, absolutely your pain will be less. (Owens et al., 2016)

Feeling a sense of connectedness to friends and family, with them sharing an understanding of living with pain and providing practical and emotional support when appropriate, was also highlighted by participants as being helpful for living a good life with pain. A participant in West et al. (2012) reported how they gained strength from the support of others: “I draw strength with the love I have for my partner, the love I get from my partner…” (West et al., 2012). A participant in Taylor (2021) reported that support from others helped them get through some of the challenges of living with pain:

I am very close to my parents, always have been—more so since my diagnosis. My family is fantastic. They are so aware of what I go through. They understand. I know if I reach out to them they have me back. I feel that way with my kids and wife. I was blessed with the people around me. (Taylor, 2021)

Participants also discussed feeling valued by others when sharing experiences and knowledge learnt from their journey with pain. Reflections included feeling purposeful and feeling helpful to others. For example:

And I like being able to help people. So I like learning different parts, like with the acupuncture… It gives me a sense of satisfaction, and I'm learning something that will help other people; it's knowledge that has been passed down through the centuries that I'm getting from somebody who has gotten that knowledge passed to him. It's kind of like the feeling that maybe you're accomplishing something that's worthwhile, not just for yourself, but for other people, and that somehow there's some good in that, that somehow being able to help another, as well as help yourself, is fulfilling in some way. (Loffer, 1999)

In contrast, the invisibility of chronic pain hindered participants from thriving. Difficulties of living with a chronic illness that is not visible to others led to participants feeling dismissed, overlooked and not believed. Participants discussed how these experiences came from interactions with professionals and non‐professionals and left them feeling unable to rely or lean on others for support. For example, one participant in Hernandez (2022) stated: “A lot of times people are like, you're not really in pain. Like, you don't look like you're in pain.” (Hernandez, 2022) and in Sheedy et al. (2017), a participant reflected that, “it's an invisible illness, and there's that constant feeling that … people don't think your pain is genuine, or they think it's all in your head.” (Sheedy et al., 2017).

Participants also discussed how not feeling believed about their pain can feel as though they are medication‐seeking, leading them to feel dismissed by health care professionals; for example: “Being treated as a pill seeker or something along those lines and just being told to go to physical therapy and, you know, almost being treated as them not being believed.” (Hernandez, 2022).

#### Ascribing meaning to life alongside pain

The final theme relates to the meaning that participants attribute to life alongside chronic pain. By taking ownership of living with chronic pain and seeing the humorous side of life, appreciating life and living it to the fullest, participants were able to thrive with chronic pain. Specifically, they discussed taking responsibility for their health and putting more effort into improving this to help them live well. For example, a participant in Loffer (1999) shared: “When I started all this I didn't think, Well, I'm taking control of my health and my spirituality and all of that. But that's what it comes down to ‐taking care of yourself actually in every way—physically, mentally, emotionally, and spiritually.” (Loffer, 1999).

Participants were able to continue to enjoy life by sharing jokes and making light of their experience of living with the condition. One participant in Shaw et al. (2020) discussed how humour is contagious and part of who they are: “I can't complain about nothing. I was always laughing, making a joke, make people laugh, and when they laugh, I laugh too.” (Shaw et al., 2020). Another discussed how humour has been key for them living well with pain and helped them during difficult times:

“I think that I have a good sense of humor, and that did not diminish being in and out of hospitals and having multiple surgeries and going through trials of mediation and side effects and all that. I still had a sense of humor [such] that everybody would think, Oh, you handle it so well.” (Loffer, 1999)

Participants discussed how living with chronic pain can cause them to reflect on their values and perspectives, appreciate what they have, identify what they are grateful for and live their life to the full. A participant in Danoff‐Burg and Revenson (2005) shared:

I think [rheumatoid arthritis] made me realize I am not invincible—throughout my earlier years I enjoyed very good health, and I took it for granted. Now look at life as a gift and try not to take all the things around me for granted.

By contrast, interpreting living with this condition as loss and feeling consumed by feelings of grief—particularly related to loss of their life prior to chronic pain or loss of their preferred future—hindered thriving when living with chronic pain. An excerpt from Loffer (1999) indicates a participant's experience of grieving their old life and feeling a burden to others:

I just would love to be able to go hiking again and do the things I used to do—go camping, go hiking, go sailing. And I can't do that yet. I'm trying to do some walking now, but even that is an effort. And I'd like to feel healthy so that I could go out and date again and find somebody. Right now I feel like how could I burden anybody with this until I feel like I have a better handle on the pain. I'm still in a fair amount of pain. Maybe some wonderful person would come along where it wouldn't matter. But right now I feel like how could I bring that on to somebody else to worry about and care for me. But I can't dwell on that because it's not there. I really think you have a finite amount of energy and you've got to think where you want to spend it: where it's going to be the most productive or helpful.

## DISCUSSION

This review addressed the question, “What characteristics and approaches enable people to thrive when living with chronic pain?*”*. Psychological understanding of the factors implicated in the experience of chronic pain has traditionally been grounded in a deficit paradigm. Most of the existent literature has focused on exploring factors associated with, or predictive of, difficulties managing chronic pain. Research exploring positive aspects of living with chronic pain for adults is emerging but qualitative findings have not yet been synthesized. Therefore, this systematic review aimed to explore adults' experiences of thriving whilst living with chronic pain not experienced as a direct result of illnesses such as cancer. Qualitative data from 17 studies were synthesized, resulting in four themes: (i) attitudes towards pain and its impact on life; (ii) behavioural strategies and openness to trying new things; (iii) perceiving love, support and connection with others; and (iv) ascribing meaning to life alongside pain. All four themes described important characteristics and approaches that helped individuals to thrive with chronic pain.

Theme 1 (attitudes) aligns with previous quantitative literature that demonstrates that individuals who are considered resilient to the impact of chronic pain have more positive pain attitudes and beliefs regarding their pain than those who are considered less resilient (Karoly & Ruehlman, 2006). ‘Resilient’ individuals were also proposed to have more positive coping styles, which fits with theme 2 (behavioural strategies); developing and being open to implementing strategies and techniques to self‐manage pain. This fits with the findings of a meta‐synthesis which highlighted that enablers to pain self‐management include feeling empowered to put self‐management strategies into practice (Devan et al., 2018). Theme 3 (connection) demonstrates how social connection with others can help individuals to thrive when living with chronic pain. Building meaningful social connections creates feelings of safeness, which in turn can facilitate exploration and resource seeking (Gilbert, 2024). Positive social experiences, including social support, also have pain‐attenuating effects (Che et al., 2018) and can aid in pain self‐management (Devan et al., 2018). Theme 4 (meaning) fits with previous quantitative literature that found that post‐traumatic growth in adults with arthritis is associated with greater appreciation of life and the ability to draw on and recognize personal strengths (Abraído‐Lanza et al., 1998; Dirik & Karanci, 2008; Purc‐Stephenson, 2014).

Seligman's PERMA model (i.e., positive emotion, engagement, relationships, meaning and accomplishments pillars; Seligman, 2011) posits that thriving results from the intersection between all five pillars, thus highlighting therapeutic trajectories that could enhance thriving and flourishing whilst living with pain. Results of the current review align well with this model, as displayed in Figure 3. Theme 1 (attitudes) appears to map onto all pillars, with attitudes impacting emotion, engagement, meaning, relationships and accomplishments, and ultimately the ability to thrive. The positive emotion pillar is congruent with themes 3 (connection) and 4 (meaning), despite pain being defined as an unpleasant sensory and emotional experience. Engagement especially links with theme 2 (behavioural strategies); that is, being open to trying new approaches and having a commitment to putting actions in place when living with chronic pain. Relationships fit with theme 3 (connection), whilst the pillar of meaning links to both themes 1 (attitudes) and 4 (meaning). Finally, accomplishments map onto several themes (attitudes, behavioural strategies, connection) which highlights the importance of engaging in valued activities, connecting with others and holding positive attitudes towards pain.

Findings from this review thus indicate that people living with chronic pain can have characteristics or develop personal approaches that facilitate thriving. For those who require therapeutic input to move towards thriving, the PERMA (Seligman, 2011) model of well‐being can be usefully extended to a chronic pain population as a therapeutic intervention to help individuals live well with pain. In particular, findings align with the core tenets of acceptance and commitment therapy (ACT; Hayes et al., 1999), which encourages the development of psychological flexibility (i.e., an individual's ability to manage, engage with, accept and adapt to difficult situations; Burton & Bonanno, 2016) and awareness and acceptance of difficulties to move forward towards a valued life. Whilst ACT is recommended in NICE (2021b) guidelines as a treatment for anxiety and depression (i.e., distress) experienced by individuals with chronic pain, the findings of the current review indicate that this approach may also be helpful in supporting individuals living with chronic pain to thrive. A recent review (McCracken, 2024) highlights that the evidence for psychological flexibility and ACT in chronic pain is large and consistently supportive. Comparatively less research has focused on the relationship between psychological flexibility and flourishing, but initial findings suggest that psychological flexibility may provide a theoretical explanation of flourishing (or not) in the presence of chronic pain (Trompetter et al., 2019).

Indeed, positive psychology interventions already draw on the PERMA model and the effectiveness of such interventions in reducing distress within chronic pain samples are well‐documented. For example, a six‐week positive psychology intervention consisting of specific exercises including Three Good Things (i.e., writing down three things that went well and reflecting on these daily; Seligman et al., 2005; Seligman et al., 2006), savouring (Bryant, 2003), self‐compassion‐focused exercises (Neff, 2009; Smeets et al., 2014) and best possible self‐imagery (i.e., imagining a life considering what it would look like if it were the best possible future to be; Meevissen et al., 2011; Peters et al., 2010) was found to reduce levels of catastrophizing (i.e., viewing a situation as worse than it is), depressed mood and anxiety in chronic primary pain patients (Flink et al., 2015). Positive psychology interventions have also been found to improve well‐being (Müller et al., 2016), with a randomized controlled trial in a chronic pain sample finding that a positive psychology intervention led to a significant increase in happiness (Peters et al., 2017). Moreover, recently published systematic reviews reported that positive psychology interventions can improve mental health in chronic pain patients (Blasco‐Belled et al., 2025; Braunwalder et al., 2022). Decreased anxiety (moderate and significant effect) and depression (small but non‐significant effect), and increased subjective well‐being (moderate and significant effect) were reported for the chronic pain sample (Blasco‐Belled et al., 2025).

### Strengths and limitations

This is the first qualitative evidence synthesis exploring the experiences of adults living with chronic pain to understand characteristics and approaches that help or hinder them to thrive. A key strength of this review is that it supports the movement within psychological research from a deficit paradigm to a focus on positive psychology, which has a direct impact on evidence‐based practice. Guidance for the conduct and reporting of systematic reviews was followed to ensure the rigour and reproducibility of this work (CRD, 2009; PRISMA; Page et al., 2021). Inclusion of grey literature aimed to address publication bias common to systematic reviews (Cherry et al., 2023), and GRADE‐CERQual was used to determine the level of confidence that can be placed in the findings. GRADE‐CERQual results indicated moderate confidence in the findings, with key methodological weaknesses identified (i.e., studies insufficiently reporting the authors' role in the study). Whilst the risk of bias has been discussed transparently when interpreting the results, we are aware of the potential for bias within this review (e.g., not all studies were dual‐screened for inclusion; Barnett‐Page & Thomas, 2009).

The findings of this review predominantly rely on the experiences of White participants living in Global North countries (as defined in Khan et al., 2022), which is reflective of the evidence synthesized and possibly the methodological decision to only include English‐language papers. Despite not specifying peer review or study location as an eligibility criterion to maximize inclusion of relevant literature, only one study was conducted in the Global South; further research in Global South countries is therefore needed to determine the applicability of these findings. Studies commonly under‐reported pain characteristics, which may limit the transferability of the findings. Furthermore, studies reflect the perspectives of a wide range of individuals living with chronic pain (i.e., those newly diagnosed to those living with pain for upwards of 50 years). Heterogeneity in pain‐related experiences may thus have influenced the conclusions that can be drawn from this review or its transferability. Whilst the data did not indicate that the time elapsed since diagnosis was linked to thriving, the processes involved in thriving can still take time to establish. As we did not specify duration of time living with chronic pain as an eligibility criterion, we therefore could not draw conclusions regarding the temporal development of thriving (i.e., how long it may take), or when the onset may be (i.e., individuals can live with chronic pain many years before receiving a diagnosis). This warrants further exploration. Furthermore, the review synthesized only the experiences of individuals living with chronic primary pain or arthritic pain, and thus, its generalizability to the experiences of individuals with secondary chronic pain requires further investigation. For example, for individuals living with chronic pain as a result of cancer, different attitudes (theme 1) regarding pain may be evident as the pain they experience is linked to a potentially life‐limiting condition. In addition, individuals living with chronic cancer pain may have different views on coping strategies depending on stage of cancer, which could in turn impact management of chronic pain beyond their control. Similarly, we did not include terms of psychological flexibility within our search. Future research may usefully explore psychological flexibility as a cognitive mechanism or process by which individuals flourish in the context of chronic pain. Finally, whilst the thematic synthesis method proposed by Thomas and Harden (2008) is a well‐used and comprehensive method of integrating qualitative research findings, there are risks of oversimplification of data (Barnett‐Page & Thomas, 2009) and interpretive power (Thomas & Harden, 2008) that should be acknowledged when interpreting findings. For example, we were only able to synthesize direct quotations presented within the included studies; access to the raw data of the included studies may have yielded different insights and resulted in alternative themes being identified and presented.

### Implications and applications

The present findings may guide health care professionals to improve how they support individuals living with chronic pain by embedding positive psychology approaches into their usual practice (see e.g., Janevic et al., 2022; Georgiadis & Johnson, 2023). In addition to problem‐focused discussion, that is, a deficit paradigm, we recommend that healthcare professionals and multidisciplinary teams (MDTs) may wish to emphasize how patients can draw on personal strengths to live well with pain. This may also shape conversations between professionals within MDTs by shifting attention away from problem‐based thinking that may not lead to therapeutic benefit (cf. Schofield, 2018).

Application of the PERMA model within psychological therapy and pain management programmes (PMP) that aim to increase coping, adjustment and living well with chronic pain could further enable individuals to thrive. Whilst more research is needed to understand how PMPs currently incorporate positive psychology principles, enquiring about positive emotion, engagement, relationships, meaning and accomplishments during assessment may shape formulations and help the psychologist and client to identify areas to target to help them to thrive. Third‐wave approaches such as ACT (already a recommended intervention for emotional distress experienced by individuals with chronic pain; NICE, 2021b), may be particularly useful in supporting this patient group to live well. It may also be beneficial to draw upon relational and systemic therapies (i.e., interventions understanding the systems in which individuals are based, how these interact and wider contextual factors such as social economic status, and access to healthcare that may influence pain and well‐being) when exploring the pillar of relationships, or to consider compassion (i.e., interventions focusing on empathy, kindness and warmth towards self, others and from others) in shaping outlook on life.

## CONCLUSION

This review identified four themes of importance in helping an individual to thrive with chronic pain: (i) attitudes towards pain and its impact on life; (ii) behavioural strategies and openness to trying new things; (iii) perceiving love, support and connection with others; and (iv) ascribing meaning to life alongside pain. These themes broadly fit with Seligman's (2011) PERMA model of thriving, supporting the utility of the model in understanding thriving in individuals living with chronic pain. A focus on acceptance and positive psychology interventions/approaches may promote thriving in adults living with chronic pain. For example, endorsing the shift away from a problem‐based narrative within MDTs and between patients and professionals can help empower individuals living with chronic pain to explore some of the positive factors and appreciation of life, identify personal values and embark on a journey focused on their goals to thrive.

## REFLEXIVITY STATEMENT (LEAD RESEARCHER)

As a Clinical Psychologist, my interest in this study stemmed from professional work experience alongside a personal curiosity about the universality of chronic pain, including the emotional pain that comes alongside it. I have been interested in chronic pain for a number of years and wished to pursue the passion to improve the evidence base in this field.

I acknowledge that for a six‐month period whilst completing this systematic review, I was working in a Facial Pain NHS Service. Whilst working within this service, my clinical practice was aligned to a process‐based therapy approach and integrated both cognitive behavioural therapy (CBT) and acceptance and commitment therapy (ACT). I acknowledge that these experiences and the knowledge I gained may have influenced my interpretation of results. However, throughout the research process, I have remained aware of potential biases, including my inclination towards CBT and ACT, as well as the terminology utilized. In addition, to make a conscious effort to help reduce biases within this research, I engaged in conversations with colleagues with different theoretical perspectives and made my own reflections throughout.

## AUTHOR CONTRIBUTIONS

**Helena Widdrington:** Conceptualization; writing – original draft; methodology; writing – review and editing; formal analysis. **Charlotte Krahé:** Supervision; conceptualization; methodology; writing – review and editing. **Katie Herron:** Conceptualization; writing – review and editing; methodology; supervision. **Kimberley Smith:** Writing – original draft; methodology; formal analysis; writing – review and editing. **Mary Gemma Cherry:** Conceptualization; methodology; writing – review and editing; supervision.

## CONFLICT OF INTEREST STATEMENT

The authors report there are no competing interest to declare.

## Data Availability

Data sharing is not applicable to this article as no new data were created or analysed in this study.
